# Pyruvate kinase M2 deregulation enhances the metastatic potential of tongue squamous cell carcinoma

**DOI:** 10.18632/oncotarget.19291

**Published:** 2017-07-17

**Authors:** Wei Wang, Qianting He, Jingjing Sun, Zhonghua Liu, Luodan Zhao, Zhiyuan Lu, Xiaofeng Zhou, Anxun Wang

**Affiliations:** ^1^ Department of Oral and Maxillofacial Surgery, First Affiliated Hospital, Sun Yat-sen University, Guangzhou, Guangdong 510080, China; ^2^ Center for Molecular Biology of Oral Diseases, Department of Periodontics, College of Dentistry, University of Illinois at Chicago, Chicago, IL 60612-7213, USA; ^3^ UIC Cancer Center, Graduate College, University of Illinois at Chicago, Chicago, IL 60612-7213, USA

**Keywords:** tongue squamous cell carcinoma, pyruvate kinase M2, metastasis, manganese superoxide dismutase, miR-138

## Abstract

Pyruvate kinase M2 (PKM2) has been verified to correlate with the prognosis of many types of cancer. However, its role in the development and metastasis of tongue squamous cell carcinoma (TSCC) remains unclear. The immunohistochemistry (IHC) results confirmed that PKM2 is overexpressed in patients with TSCC. PKM2 up-regulation was related to lymph node metastasis and associated with reduced overall survival. According to the microarray analysis and Western blots, PKM2 expression was up-regulated in TSCC cells with enhanced metastatic potential. PKM2 knockdown inhibited cell migration and invasion, reduced SOD2 (manganese superoxide dismutase) activity and the intracellular H_2_O_2_ level, and inhibited tumour growth and lung metastasis *in vivo*. PKM2 overexpression promoted cell migration and invasion, and increased SOD2 activity and the intracellular H_2_O_2_ level. Moreover, miR-138 directly targeted PKM2 and inhibited PKM2 expression. Thus, PKM2 deregulation plays an important role in TSCC and may serve as a biomarker of metastatic potential or as a therapeutic target in patients with TSCC. PKM2, a miR-138 target gene, enhances the metastatic potential of TSCC through the SOD2-H_2_O_2_ pathway.

## INTRODUCTION

In contrast to normal cells, which rely primarily on mitochondrial oxidative phosphorylation to generate energy, most cancer cells rely on glycolysis, even in conditions of normal oxygen concentration; this metabolic condition is termed the Warburg effect [1]. Pyruvate kinase (PK) is the enzyme involved in the final step of glycolysis, catalysing the dephosphorylation of phosphoenolpyruvate (PEP) to pyruvate and generatingan adenosine triphosphate (ATP) molecule [2]. Four isoforms of PK (PKM1, PKM2, PKL and PKR) have been identified, and the expression of these isoforms is tissue dependent. PKM2 is encoded by the PKM gene and is expressed in embryonic cells, adult stem cells, and cancer cells [3]. PKM2 gene transcription is controlled by many factors [4–8]. For example, hypoxia-inducible factor (HIF)-1 activates PKM2 gene transcription in hypoxic cells by binding to a hypoxia response element located within the first intron of the PKM2 gene [4]. Myc regulates PKM2 expression both directly, by binding to a Myc response element located in the promoter of the PKM2 gene, and indirectly, by activating the transcription of genes encoding hnRNPI, hnRNPA1, and hnRNPA2, which facilitate the alternative splicing that generates PKM2 mRNA [5, 6]. Some microRNAs (miRNAs), such as miR-326 [7], miR-133a and miR-133b [8], have also been found to directly target and regulate the synthesis of PKM2 protein [7, 8].

In addition to its role as a PK, PKM2 also functions as a protein kinase and as a transcriptional coactivator [9]. Many studies have reported that cancer tissues exclusively express PKM2 and that PKM2 is important for cancer metabolism and tumour growth, invasion and metastasis [2, 10, 11]. Enhanced PKM2 expression has been observed both in various cancer cell lines and in blood, serum, and stool samples from cancer patients [3]. PKM2 knockdown decreases *in vitro* cell proliferation and glucose metabolism and inhibits the growth of xenografts [2]. To date, few studies have focused on the relationship between PKM2 and TSCC [8, 12]. Moreover, the intracellular events elicited by PKM2 are far more complicated and require further investigation.

TSCC is most common carcinoma in the oral and maxillofacial region and is characterized by rapid local invasion and migration. Our previous studies [13–20] demonstrated that the abnormal expression of manganese superoxide dismutase (SOD2), miR-138, miR-222 and miR-181a can influence TSCC invasion and metastasis through different signalling pathways, such as the miR-138-Slug, the SOD2-H_2_O_2_ and the extracellular signal-regulated kinase (ERK)-Slug pathways. To further investigate the role of PKM2 in the development and metastasis of TSCC and elucidate whether PKM2 enhances the metastatic potential of TSCC via the above relevant pathways (the SOD2-H_2_O_2_ and miR-138 pathways), we detected the expression of PKM2 in TSCC by immunohistochemistry (IHC). Then, we investigated the role of PKM2 in the migration and invasion of TSCC cells *in vitro* and *in vivo*. Finally, we analysed the mechanism of how PKM2 mediated the metastatic potential of TSCC. We found that the deregulation of PKM2 plays an important role in the progression of TSCC. PKM2, a miR-138 target gene, enhances the metastatic potential of TSCC through the SOD2-H_2_O_2_ pathway.

## RESULTS

### PKM2 deregulation is associated with the development and prognosis of TSCC

**Figure 1:**
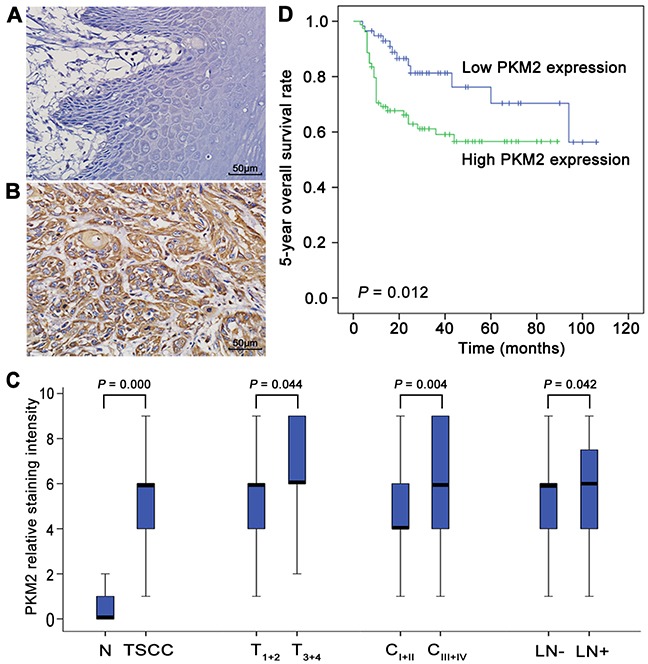
PKM2 deregulation in the development of TSCC and patient prognosis PKM2 expression in normal tongue tissues **(A)** and TSCC tissues **(B)** was analysed by IHC. **(C)** Box plots present a comparison of PKM2 expression in normal tongue tissue samples and tissue samples from TSCC cases with different tumour stages, different clinical stages, and with or without lymph node metastasis. Each box represents the 25th to the 75th percentile of the observations, and the line in the middle of the box represents the median. **(D)** Kaplan–Meier plots of the 5-year OS in patient groups as defined by PKM2 expression. The differences in survival rates are statistically significant between the high and low PKM2 expression groups (*P=*0.012). The numbers of cases in the high PKM2 expression group and low PKM2 expression group were 79 and 58, respectively. Scale bar: 50 μm.

As illustrated in Figure 1A-1B, PKM2 was expressed in the cytoplasm in TSCC tissues and was barely detectable in normal tongue tissue samples. The expression of PKM2 was significantly increased in the primary cancer tissues compared with the normal tongue tissues (Figure 1C). Among the TSCC cases, the levels of PKM2 expression were significantly higher in T_3+4_ than in T_1+2_, in C_III+IV_ than in C_I+II_, and in cases with lymph node metastasis than in cases without lymph node metastasis (Figure 1C). No significant differences were found in PKM2 expression with respect to age, gender or pathological grade (Supplementary Table 1).

To elucidate the prognostic role of PKM2 expression in patients with TSCC, we examined the relationship between PKM2 expression and patient outcome using long-term follow-ups. As illustrated in Figure 1D, a striking difference was observed in the 5-year overall survival (OS) between the high and low PKM2 expression groups (*P=*0.012). The survival period of the high PKM2 expression group was shorter than that of the low PKM2 expression group (mean survival periods, 56.4 vs 81.5 months). To further evaluate the association of the PKM2 expression level and clinicopathological factors with the prognosis of TSCC patients, univariate and multivariate analyses were conducted. As shown in Supplementary Table 2, both the univariate and multivariate analyses indicated that the PKM2 expression level is a prognostic factor for the 5-year OS of TSCC patients.

### PKM2 expression is related to the migratory/invasive ability of TSCC cells

To investigate whether PKM2 is related to TSCC cell migration/invasion, at first, gene expression profiling was performed by microarray analysis on three pairs of TSCC cell lines with different migration/invasion abilities. As shown in the gene ontology (GO) analysis presented in Supplementary Table 3, 33 genes were associated with glycolysis (GO: 0006096), and PKM2 was consistently down-regulated in less migratory/invasive TSCC cells (UM2 cells, miR-138 mimic-transfected UM1 cells or SCC9 cells) compared with more migratory/invasive TSCC cells (UM1 cells, control mimic-transfected UM1 cells or SCC9 cells) [16]). PKM2 protein level detected by western blot was also found to be increased in the more migratory/invasive UM1 cells compared with the less migratory/invasive UM2 cells (Supplementary Figure 1).

### PKM2 regulates TSCC cell migration/invasion through the SOD2-H_2_O_2_pathway

**Figure 2:**
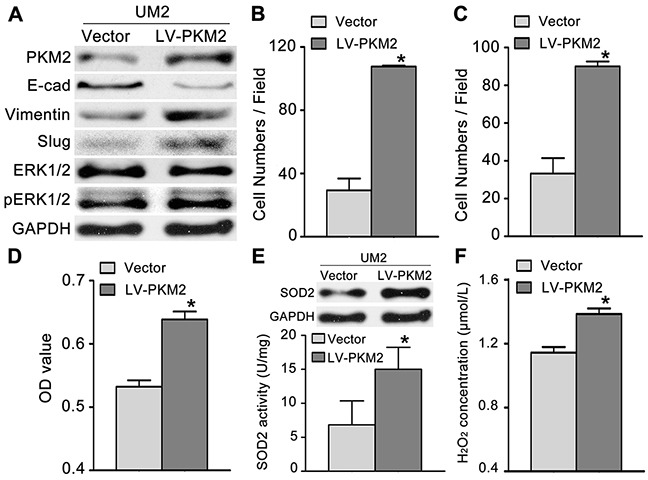
PKM2 overexpression promotes TSCC cell migration and invasion **(A)** Western blot analysis showing the levels of the PKM2, pERK, ERK and EMT marker (E-cad, Vimentin, and Slug) proteins in UM2 cells infected with a lentiviral construct containing the PKM2 cDNA (LV-PKM2) or with the control lentiviral construct (Vector). PKM2 overexpression promoted UM2 cell migration **(B)** and invasion **(C)**. PKM2 overexpression also resulted in increased UM2 cell proliferation **(D)**, SOD2 expression and activity **(E)** and H_2_O_2_ production **(F)**. *P<0.05.

To investigate the role of PKM2 in TSCC cell migration/invasion, a pair of TSCC cell line, UM1 and UM2 cell line, was used, in which UM1 has higher migration/invasion abilities than UM2 [16]. UM2 cells with lower PKM2 expression (Supplementary Figure 1) were infected with a lentiviral construct containing the PKM2 cDNA (lenti-PKM2). The protein concentration of PKM2 was increased in UM2 cells after infection with lenti-PKM2 (Figure 2A). UM2 cells transfected with lenti-PKM2 exhibited increased migration (Figure 2B) and invasion (Figure 2C) compared with cells infected with the control lentivirus. PKM2 overexpression in UM2 cells also resulted in increased proliferation (Figure 2D), increased SOD2 expression and activity (Figure 2E) and increased H_2_O_2_ production (Figure 2F), but no changes in Catalase expression or activity were observed (Supplementary Figure 2A). The levels of pERK1/2 (phosphorylated ERK1/2) and the EMT (epithelial–mesenchymal transition) markers (Slug and Vimentin) were clearly increased, and E-cadherin expression was decreased as a result of PKM2 overexpression in UM2 cells. PKM2 overexpression in UM2 cells did not obviously change the ERK1/2 levels (Figure 2A).

**Figure 3:**
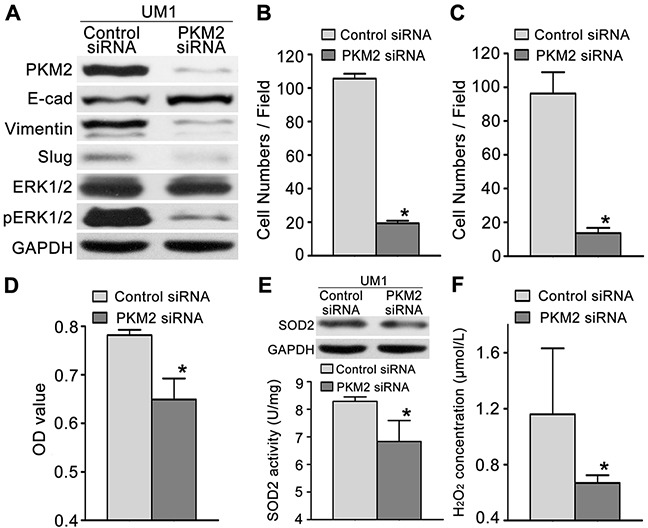
PKM2 knockdown inhibits TSCC cell migration and invasion **(A)** Western blot results showing the levels of the PKM2, pERK, ERK and EMT marker (E-cad, Vimentin, and Slug) proteins in UM1 cells transfected with the PKM2 siRNA or control siRNA. PKM2 knockdown inhibited the migration **(B)** and invasion **(C)** of UM1 cells. PKM2 knockdown also inhibited UM1 cell proliferation **(D)**, SOD2 expression and activity **(E)** and H_2_O_2_ production **(F)**. *P<0.05.

Furthermore, we knocked down PKM2 expression in UM1 cells (higher PKM2 expression) using RNA interference. The protein levels of PKM2 were significantly decreased in UM1 cells after transfection with PKM2 siRNA (Figure 3A). UM1 cells transfected with PKM2 siRNA displayed decreased migration (Figure 3B) and invasion (Figure 3C) compared with the control siRNA-transfected cells. PKM2 knockdown in UM1 cells also reduced proliferation (Figure 3D), SOD2 expression and activity (Figure 3E) and H_2_O_2_ production (Figure 3F), but no changes in Catalase expression and activity were observed (Supplementary Figure 2B). Moreover, PKM2 knockdown also decreased the expression levels of pERK1/2 and the EMT markers (Slug and Vimentin) and increased the E-cadherin expression level, but no obvious changesin the ERK1/2 levels were detected (Figure 3A).

### The miR-138-PKM2 pathway regulates TSCC cell migration and invasion

**Figure 4:**
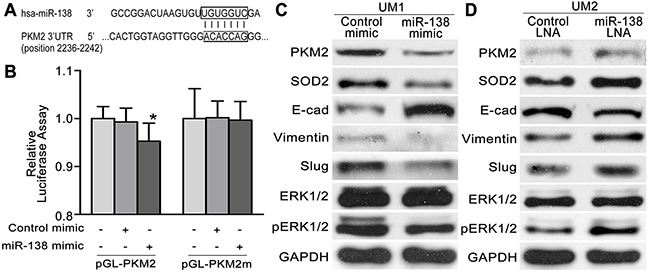
The miR-138-PKM2pathwayregulates TSCC cell migration/invasion **(A)** Predicted target sequences for miR-138 are located in the 3’-UTR of PKM2 mRNA. **(B)** Dual luciferase reporter assays were performed to evaluate the target genes of miR-138. Following transfection with pGL-PKM2 for 24 h, luciferase activity was significantly reduced in cells treated with miR-138 mimics relative to cells treated with control mimics (*P<0.05 vs blank). After the seed region of the target site was mutated (pGL-PKM2 m), the effects of miR-138 on luciferase activity were abolished. **(C, D)** Western blot results showing the levels of the PKM2, SOD2, pERK, ERK and EMT marker (E-cad, Vimentin,and Slug) proteins in TSCC cells transfected with the miR-138 mimic (C) or miR-138 LNA (D).

Our previous studies revealed that the miR-138 pathway plays an important role in the migration and invasion of TSCC cells [14–16, 21, 22]. Based on the bioinformatics analysis, a targeting site for hsa-miR-138 was identified in the 3’-UTR of PKM2 mRNA (Figure 4A). As illustrated in Figure 4B, dual luciferase reporter assays confirmed that miR-138 directly targets PKM2. When cells were co-transfected with miR-138 mimics and luciferase reporter constructs carrying pGL-PKM2, luciferase activity was significantly reduced. However, the effect of miR-138 on luciferase activity was abolished when the seed regions of the targeting sites were mutated (pGL-PKM2 m). To further confirm the role of the miR-138-PKM2 pathway in TSCC, a functional analysis was performed. When UM1 cells were transfected with the miR-138 mimic, which decreased the migration and invasion of UM1 cells [16], the expression levels of PKM2, SOD2, pERK1/2, Slug and Vimentin were obviously reduced, whereas E-cadherin expression was increased (Figure 4C). In contrast, when UM2 cells were transfected with the miR-138 LNA, which increased the migration and invasion of UM2 cells [16], the expression levels of PKM2, SOD2, pERK1/2, Slug and Vimentin were obviously increased, whereas E-cadherin expression was obviously decreased (Figure 4D).

### PKM2 knockdown inhibits tumour growth and lung metastasis *in vivo*

**Figure 5:**
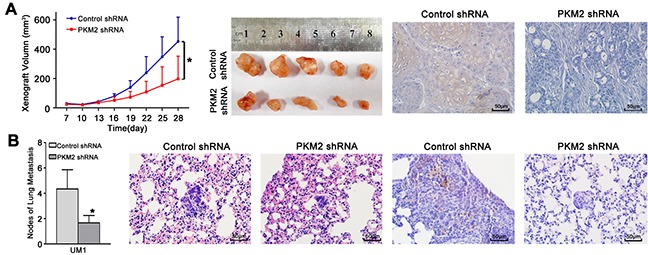
PKM2 knockdown inhibits TSCC xenograft growth and lung metastasis *in vivo* **(A)** CAL27 cells stably infected with lentiviral constructs containing control shRNA or PKM2 shRNA were subcutaneously injected into nude mice. Xenografts were measured every 3 days with a calliper, and the resulting growth curves demonstrated that PKM2 knockdown significantly inhibited CAL27 xenograft growth. **(B)** UM1 cells stably infected with lentiviral constructs containing control shRNA or PKM2 shRNA were injected into the tail vein of nude mice. TSCC metastasis to the lung was assessed 8 weeks after injection. The histopathological analysis of lung metastasis (magnification 400×) and metastatic lung nodules are shown. PKM2 expression in xenografts (A) and lung metastases (B) was analysed using IHC. Scale bar: 50 μm. *P<0.05.

To further confirm the role of PKM2 in TSCC growth and metastasis *in vivo*, the growth and lung metastases of xenografts in nude mice were examined. CAL27 cells, which showed higher PKM2 expression (Supplementary Figure 1) and migration/invasion ability similar to UM1 cells [19], were stably infected with lentiviral constructs containing PKM2 shRNA (lenti-PKM2 shRNA) or control shRNA (lenti-control shRNA) and were then subcutaneously injected into nude mice. As shown in Figure 5A, tumour growth was significantly suppressed in the group treated with lenti-PKM2 shRNA compared with the group treated with lenti-control shRNA, with an inhibition rate on day 28 of 54.9%. The tumour doubling times were 1.4 days (control shRNA) and 2.3 days (PKM2 shRNA). PKM2 expression was obviously decreased in xenografts from the group treated with the lenti-PKM2 shRNA compared with the group treated with the lenti-control shRNA. UM1 cells stably infected with lenti-PKM2 shRNA were also injected into the tail veins of nude mice, and metastatic nodules in the lungs were confirmed histologically and counted as in our previous study [19]. Mice injected with the lenti-PKM2 shRNA-transfected cells exhibited a significantly reduced number of metastatic nodules compared to mice injected with the lenti-control shRNA-transfected cells (Figure 5B). PKM2 expression was obviously decreased in the metastatic nodules from the group treated with the lenti-PKM2 shRNA compared with the group treated with the lenti-control shRNA.

## DISCUSSION

Among the glycolytic isozymes, the expression of PKM2 particularly favours aerobic glycolysis and cell proliferation due to the intracellular localization and kinetic properties [23]. To date, many studies have revealed high PKM2 expression levels in cancer cells, which were associated with metastasis and poor prognosis in cancer patients [9, 24, 25]. Recently, PKM2 deregulation has also been identified during the carcinogenesis of oral squamous cell carcinoma and TSCC [12, 26]. In this study, we found that PKM2 deregulation was a frequent event in the progression of TSCC and that PKM2 up-regulation was not only related to lymph node metastasis but also a prognostic factor in patients with TSCC. Moreover, we found that TSCC cells with higher migration/invasion capabilities had higher PKM2 expression levels. PKM2 overexpression promoted the proliferation, migration and invasion of TSCC cells, increased the expression of EMT markers (Slug and Vimentin) and decreased the expression of E-cadherin; whereas PKM2 knockdown inhibited the proliferation, migration and invasion of TSCC cells *in vitro* and *in vivo*, decreased the expression of EMT markers and increased the expression of E-cadherin. Based on these findings, PKM2 plays an important role in the development and prognosis of TSCC. PKM2 also regulates the proliferation and metastasis of TSCC *in vitro* and *in vivo*.

**Figure 6:**
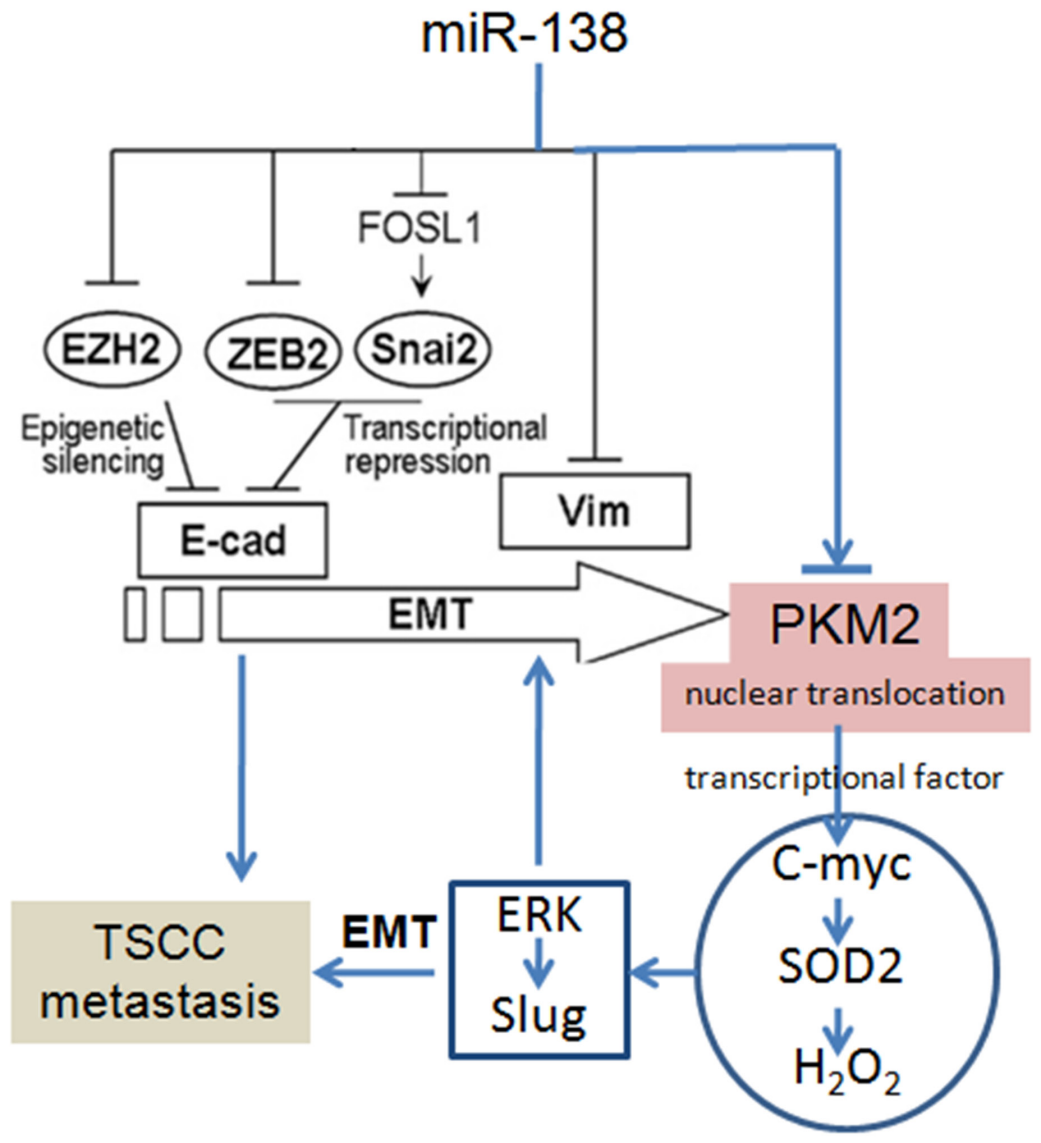
PKM2 regulates TSCC cell migration/invasion through miR-138 and the SOD2-H_2_O_2_ pathway The pathways for miR-138-regulated EMT had been revealed in our previous reports [14–16, 21, 22, 27].

Many factors have been found to regulate PKM2 gene transcription, such as miRNAs [7–9]. Kefas*et al.* found that miR-326 matches two regions in the 3’-UTR of PKM2 mRNA and that the transfection of the miR-326 precursor decreased both PKM2 3’-UTR-luciferase reporter activity and PKM2 protein levels in glioma cells [7]. Based on the bioinformatics analysis, a targeting site for miR-138 was also identified in the 3’-UTR of the PKM2 mRNA. As shown in our previous studies, miR-138 is frequently down-regulated in TSCC. Statistically significant reductions in the miR-138 level were observed in 13 out of 15 TSCC samples tested compared to their matching control samples, and in all 7 TSCC cell lines compared to normal human oral keratinocytes (NHOK). The miR-138 pathway plays an important role in the migration and invasion of TSCC cells [14–16, 21, 22]. miR-138 regulates the EMT via multiple distinct pathways, including directly targeting the Vimentin mRNA or the transcriptional repressors ZEB2 and Slug, which in turn regulates the transcription of the E-cadherin gene [14–16, 21, 22, 27]. In the present study, we also verified that miR-138 directly targeted PKM2. Overexpression of miR-138 resulted in decreased PKM2 expression, whereas the inhibition of miR-138 increased PKM2 expression. Moreover, miR-138 down-regulation was accompanied by a marked reduction in E-cadherin expression and increased Vimentin expression, which are characteristics of the EMT. According to the study by Hamabe and colleagues, stimulation of the EMT promotes the nuclear translocation of PKM2 in colon cancer cells [28], and nuclear PKM2 acts as an active protein kinase or transcriptional factor, thereby conferring an enhanced malignant potential to the cells [3, 28]. Based on these results, PKM2 has the potential to enhance the migration/invasion potential of TSCC cells may through the miR-138-PKM2 pathway, as miR-138 directly targets PKM2 and the miR-138-mediated EMT results in the nuclear translocation of PKM2 (Figure 6).

In addition to its role as a PK, PKM2 also functions as a protein kinase and a transcriptional coactivator. PKM2 stimulates the transcriptional activities of HIF, β-catenin, STAT3, and Oct4 [24]. PKM2 also stimulates the epidermal growth factor-induced transcription of the β-catenin target gene C-myc [9]. In our previous studies, we showed that SOD2 is a direct target gene of C-myc, and we demonstrated C-myc-SOD2-mediated TSCC cell migration and invasion. Moreover, SOD2-dependent H_2_O_2_ production contributed to the migration and invasion of TSCC and salivary adenoid cystic carcinoma cells via the ERK-Snail (Slug) signalling pathway [17, 18, 29–33]. The Slug gene is an EMT transcriptional factor and plays an important role in the EMT [15]. ERK directly targets the Slug promoter and induces Slug expression and the EMT [30]. In the present study, we also found that PKM2 knockdown resulted in reduced SOD2 activity, intracellular H_2_O_2_ levels, and pERK1/2 and Slug expression, whereas PKM2 overexpression increased SOD2 activity, intracellular H_2_O_2_ levels, and pERK1/2 and Slug expression. Thus, PKM2 activate the SOD2-H_2_O_2_ pathway mainly through C-myc and indirectly activates the ERK-Slug pathway through the Myc-SOD2-H_2_O_2_ pathway. These pathways play an important role in the PKM2-mediated EMT and migration/invasion potential of TSCC (Figure 6).

As shown in this study, the deregulation of PKM2 plays an important role in the development and metastasis of TSCC and is associated with a poor prognosis for patients with TSCC. PKM2 expression contributed to increased TSCC aggression *in vitro* and *in vivo*. PKM2, a miR-138 target gene, enhanced the migration/invasion potential of TSCC cells through the SOD2-H_2_O_2_ pathway. These findings provide novel insights into PKM2 deletion as a potential therapeutic intervention for TSCC patients.

## MATERIALS AND METHODS

### Patient samples

We conducted a retrospective cohort study using data from the First Affiliated Hospital of Sun Yat-sen University. All the patients were diagnosed with TSCC and underwent radical dissection without preoperative chemotherapy or radiotherapy. The clinical characteristics of the patients are summarized in Supplementary Table 4. These TSCC and 20 adjacent normal tongue tissue samples from the TSCC patients were used to assess PKM2 expression by IHC. Survival was calculated from the time of diagnosis to the date of death or the date of the latest follow-up visit (2014-09-01). This study was approved by the Institute Research Ethics Committee of the First Affiliated Hospital of Sun Yat-sen University (2016074).

### Immunohistochemical staining

IHC was used to detect PKM2 staining as previously described [13]. In brief, the sections were stained with an anti-PKM2 antibody (Cell Signalling Technology, 1:1000) and incubated overnight at 4°C. The sections were then processed using a MaxVision™ HRP-Polymer Anti-Rabbit IHC Kit (Maixin, Fuzhou, China), developed with a DAB Horseradish Peroxidase Colour Development Kit (Maixin, Fuzhou, China) and counterstained with haematoxylin. The degree of immunostaining was scored according to both the proportion of positively stained tumour cells (0∼3) and the staining intensity (0∼3), as previously described [29]. The staining index was calculated by multiplying the staining intensity score by the proportion of positive tumour cells, and the results were 0, 1, 2, 3, 4, 6, and 9. An optimal cut-off value (median) was identified as follows: a staining index score of >4 was used to define high PKM2 expression, and a score ≤4 was defined as low PKM2 expression.

### Cell culture and transfection

Human TSCC UM1, UM2, CAL27, and SCC9 cells were maintained in Dulbecco’s Modified Eagle’s Medium (Invitrogen, CA, USA) supplemented with 10% foetal bovine serum, 1,000 U/ml penicillin and 500 mg/ml streptomycin in a 37°C incubator with 5% CO_2_. The UM1 and UM2 cell lines are paired lines from a single patient with TSCC that exhibit different migration/invasion abilities [34]. Indeed, UM1 cells exhibit greater migration/invasion abilities than UM2 cells [16]. CAL27, another TSCC cell line, exhibits greater migration and invasion abilities than UM2 cells, but lower migration and invasion abilities than UM1 cells [19].

For PKM2 knockdown, the cells were seeded in 6-well plates and transfected with PKM2 small interfering RNA (siRNA) or control siRNA (Ribobio, Guangzhou, China) using Lipofectamine™ RNAiMAX transfection reagent (Invitrogen, CA, USA) according to the manufacturer’s instructions. Three sequences of PKM2 siRNA were used; then, the sequence that exhibited the best knockdown effect was chosen for further experiments. To overexpress PKM2, a lentiviral construct containing PKM2 complementary DNA (cDNA) (NM_002654.5) (Chemgene, Shanghai, China) was packaged using the ViraPower Mix (Invitrogen, CA, USA) in 293T cells. Lentiviral infection (multiplicity of infection=1:50) was performed in cells in the presence of 5 μg/ml polybrene (Sigma-Aldrich, MO, USA). miR-138 mimics, control mimics, a locked nucleic acid (LNA) inhibitor of miR-138 (anti-miR-138 LNA) and control LNA (GenePharma, Shanghai, China) were also transfected into the appropriate cells. The cells were harvested for functional analysis after 48 h. The sequences of the PKM2 siRNA, the miR-138 mimic and the miR-138 LNA are shown in Supplementary Table 5.

### *In vitro* cell migration/invasion assay

Transwell assays were performed to assess cell migration and invasion using BD BioCoat Control Cell Culture Inserts and BD BioCoat BD Matrigel^TM^ Invasion Chambers, respectively [30]. In brief, cells were seeded in the upper Boyden chambers of the cell culture inserts. After a 24 h incubation, cells that had adhered to the lower membrane were stained with 4’,6-diamidino-2-phenylindolein the dark, imaged and counted. Three random fields were captured at 200× magnification under a microscope. The number of cells on the bottom surface was compared among the groups.

### Cell proliferation assay

Cell proliferation was detected 24 h later using a modified Cell Counting Kit 8 (CCK-8) assay (Fanbo, Beijing, China) according to the manufacturer’s instructions. In brief, cells were seeded in 96-well plates at a density of 5×10^3^ cells per well. Then, 10 μl of CCK-8 solution was added to each well of the plate, which was then incubated for 2 h. The absorbance (optical density, OD) value of each well was determined at a wavelength of 450 nm using a plate reader.

### Western blot analysis

Western blot analyses were performed as previously described [17] using specific antibodies against PKM2, E-cadherin, Vimentin, Slug (member of the Snail family), ERK1/2, pERK1/2, SOD2, GAPDH (Cell Signalling Technology, MA, USA), and Catalase (Sigma-Aldrich, MO, USA). GAPDH (Cell Signalling Technology) was used as a control.

### SOD2/Catalase activity and intracellular H_2_O_2_ concentration [17]

SOD2 activity was determined using a Cu/Zn-SOD and Mn-SOD Assay Kit with WST-8 (2-(2-methoxy-4-nitrophenyl)-3-(4-nitrophenyl)-5-(2,4-disulfophenyl)-2H-tetrazolium, monosodium salt) (Beyotime, China) according to the manufacturer’s instructions. Catalase activity was detected using a Catalase Analysis Kit (Molecular Probes, USA) according to the manufacturer’s instructions. The H_2_O_2_ concentration was determined using a PeroXOquant Quantitative Peroxide Assay Kit (Pierce, IL, USA) according to the manufacturer’s instructions.

### Dual luciferase reporter assay

A dual luciferase reporter assay was performed as previously described to test whether miR-138 directly targets PKM2 [30]. In brief, the luciferase reporter gene constructs for PKM2 (pGL-PKM2) were generated by cloning the 3’-untranslated region (UTR) of PKM2 [NM_002654.5] containing the miR-138 binding site into the XhoI and NotI sites of a pGL-3-Control firefly luciferase reporter vector (Promega, Wisconsin, USA). The corresponding mutant construct (pGL-PKM2 m) was generated by replacing the seed regions of miR-138. These constructs were then verified by sequencing. The cells were transfected with the reporter constructs using Lipofectamine 2000 (Invitrogen, CA, USA). The pRL-TK vector (Promega, Wisconsin, USA) was also co-transfected and served as an internal control to normalize transfection efficiency. Luciferase activity was then determined using a GloMax 20/20 Luminometer (Promega, Wisconsin, USA).

### Detection of gene expression profiles using a microarray analysis

Three pairs of TSCC cell lines with different migration/invasion abilities were used to detect the mRNA expression profiles. SCC9 and UM1 cells transfected with the miR138 mimic have been shown to exhibit lower migration/invasion abilities than SCC9 and UM1 cells transfected with a control mimic [16]. UM1 displays greater migration/invasion abilities than UM2 cells [16]. For transfection, SCC9 or UM1 cells were plated in 6 cm diameter cell culture dishes and grown to 60% confluence. Then, 7.5 μl of the miR-138 mimic (20 μM) or control mimic (20 μM) and 6 μl of Transfection Reagent were added to 750 μl of antibiotic-free Opti-MEM perdishand mixed together to form the transfection complex. The transfection complex (100 nM) was added to cells and incubated for 24 h before replacing the medium. Cells were harvested 48 h after transfection. Total RNA was isolated, labelled, and hybridized to the Affymetrix Human Genome U133 Plus 2.0 GeneChip arrays according to standard, previously reported protocols [21, 35]. The arrays were scanned with a GeneChip Scanner 3000. The scanned array images were processed with GeneChip Operating software (GCOS). The microarray data were pre-processed using Robust Multi-array Analysis (RMA). Experiments were performed in duplicate. The differentially expressed genes were defined as those with a fold change<0.67 (down-regulated) or >1.5 (up-regulated) and a p value<0.05. The microarray data were present in Supplementary Files 1.1 and 1.2. A GO analysis was used to analyse the microarray data.

### Tumourigenesis and metastasis assays in nude mice

UM1 or CAL27 cells were stably infected with lentiviral constructs containing a PKM2 short hairpin RNA (shRNA) or control shRNA (GeneChem, Shanghai, China) using the method described in our previous study [19] to investigate the effects of PKM2 on tumourigenesis and metastasis *in vivo*. For the tumourigenesis assays, treated CAL27 cells (5×10^6^/0.2 ml) were subcutaneously injected into the flanks of 4-week-old female BALB/c nude mice (purchased from the Institute of Zoology, Chinese Academy of Sciences, Guangzhou), and the resulting xenografts were measured with a calliper beginning at 1 week after inoculation. Tumour volumes were calculated as ½length×width^2^, and the tumour growth curves (y=Ae^kday^) and tumour doubling times (ln2/k) were obtained using previously described methods [19]. For the metastasis assays, treated UM1 cells (1×10^6^/0.2 ml) were injected into the tail veins of the BALB/c nude mice. The animals were sacrificed after 8 weeks, and the metastatic tumours in the lungs were assessed as previously described [19]. No mice showed any notable toxic effects or weight loss during the experiment. 5 mice were used in each group for the above animal study. The animal study was also approved by the ethics committee of our institution (2016047).

### Statistical analysis

All statistical analyses were performed using the Statistical Package for the Social Sciences (SPSS, Chicago, IL), Version 19.0. The χ^2^ test was used to analyse the relationship between gene expression and the clinicopathological characteristics. Survival curves were plotted using the Kaplan–Meier method and were compared with the log-rank test. Cox regressions were used for the univariate and multivariate analyses. The data were analysed with Student’s t tests to determine the significance between two groups or with a one-way analysis of variance to calculate significance among more than two groups. In all cases, *P*<0.05 was considered statistically significant.

## SUPPLEMENTARY MATERIALS FIGURES AND TABLES







## Abbreviations

PKM2: pyruvate kinase M2
TSCC: tongue squamous cell carcinoma
OSCC: oral squamous cell carcinoma
SOD2: manganese superoxide dismutase
IHC: immunohistochemistry
miRNA: microRNA
ERK: extracellular signal-regulated kinase
E-cad: E-cadherin
Vim: Vimentin
EMT: epithelial–mesenchymal transition.
