# Supplementary material for: Pyruvate kinase M2 deregulation enhances the metastatic potential of tongue squamous cell carcinoma
Source: Oncotarget. 2017 Jul 17;8(40):68252–62. doi: 10.18632/oncotarget.19291 (PMC5620253; doi:10.18632/oncotarget.19291)
Supplement: Supplementary file 1 [file oncotarget-08-68252-s001.pdf]

Pyruvate kinase M2 deregulation enhances the metastatic potential of tongue squamous cell carcinoma

SUPPLEMENTARY MATERIALS

Highlights

1. PKM2 plays an important role in the development and prognosis of TSCC.

2. PKM2 enhances the migration and invasion ability of TSCC cells *in vitro*.

3. PKM2 enhances the proliferation and metastatic potential of TSCC *in vivo*.

4. PKM2 regulates the metastatic potential of TSCC via the C-myc-SOD2-H_2O_2 pathway.

5. PKM2 regulates the metastatic potential of TSCC via the miR-138-PKM2 pathway.

Supplementary Table 1: The relationship between PKM2 expression and the clinicopathological characteristics of TSCC patients

Characteristic		PKM2		*P* value
		Low	High	
Gender	Male	40	49	0.400
	Female	18	30	
Age	≤40	7	11	0.751
	>40	51	68	
Tumour stage	T_{1+2}	49	50	0.006
	T_{3+4}	9	29	
LN metastasis	Negative	46	47	0.014
	Positive	12	32	
Clinical stage	C_{I+II}	43	37	0.001
	C_{III+IV}	15	42	
Pathological grade	Well	30	29	0.079
	Moderate/Poor	28	50	

LN: Lymph node.

Supplementary Table 2: Results of the univariate and multivariate analyses of factors affecting the 5-year overall survival of patients with TSCC

Characteristic	Univariate analysis	Multivariate analysis		
	P value	*P* value	HR	95% CI
Age (>40 vs ≤40)	0.275	0.056	2.879	(0.974, 8.509)
Gender (Female vs Male)	0.258	0.063	1.912	(0.967, 3.783)
Pathological grade (Moderate/ Poor vs Well)	0.003	0.006	2.827	(1.352, 5.909)
Tumour stage (T_{1+2} vs T_{3+4})	0.001	0.012	3.360	(1.309, 8.628)
LN metastasis (Positive vs Negative)	0.022	0.044	2.086	(1.020, 4.266)
Clinical stage (C_{I+II} vs C_{III+IV})	0.007	0.539	0.734	(0.274, 1.967)
DM (Positive vs Negative)	0.519	0.309	0.565	(0.188, 1.696)
PKM2 expression (Low vs High)	0.012	0.044	2.148	(1.020, 4.521)

Supplementary Table 3: Glycolysis (GO: 0006096)-related gene expression patterns in TSCC cells with different metastatic potentials determined by microarray analysis

Gene and Name	SCC-9 miR138 mimic/ control mimic	UM1 miR138 mimic/ control mimic	UM2/UM1
ADPGKA DP-dependent glucokinase	0.6869	0.6522	0.4922
ALDOA aldolase A, fructose-bisphosphate	0.7322	0.9640	0.8609
HK1 hexokinase 1	0.3618	0.7103	0.5726
HK2 hexokinase 2	0.2495	0.5670	0.4955
TPI1 triose phosphate isomerase 1	0.3454	0.7377	0.6186
LDHB lactate dehydrogenase B	0.8548	0.6770	0.6357
PKM2 pyruvate kinase, muscle	0.3601	0.6528	0.7956
MDH2 malate dehydrogenase 2, NAD (mitochondrial)	0.3386	0.8062	0.6388
DHTKD1 dehydrogenase E1 and transketolase domain containing 1	0.2617	0.8891	0.6093
HKDC1 hexokinase domain containing 1	0.7809	0.8024	0.6306
ALDOB aldolase B, fructose-bisphosphate	2.0332	2.8690	1.2333
ALDOC aldolase C, fructose-bisphosphate	1.0501	1.5333	1.0131
ENO2 enolase 2 (gamma, neuronal)	1.0428	1.1432	2.5987
PFKP phosphofructokinase, platelet	1.0719	1.1651	1.1925

*: SCC9 and UM1 cells stably transfected with the miR138 mimic have been shown to have lower migration/invasion abilities than SCC9 and UM1 cells transfected with a control mimic [16].

Supplementary Table 4: Clinicopathological characteristics of patients with TSCC*

		TSCC	Normal
Gender	Male: n (%)	89 (64.96)	11 (55.00)
	Female: n (%)	48 (35.04)	9 (45.00)
Age	Median (range)	57 (27–84)	52 (37–78)
Tumour stage	T_{1+2}: n (%)	100 (72.99)	
	T_{3+4}: n (%)	37 (27.01)	
LN metastasis	Negative: n (%)	93 (67.88)	
	Positive: n (%)	44 (32.12)	
Clinical stage	C_{I+II}: n (%)	80 (58.39)	
	C_{III+IV}: n (%)	57 (41.61)	
Pathological grade	Well: n (%)	59 (43.07)	
	Moderate/Poor: n (%)	78 (56.93)	

*: No distant metastasis was detected. Therefore, M0/M1 data are not available in this study.

Supplementary Table 5: Sequences of the PKM2 siRNA and miR-138

	Sequence
PKM2 siRNA	5'-CCAUAAUCGUCCUCACCAATdT-3'
Control siRNA	5'-UUCUCCGAACGUGUCACGUTT-3'
Control mimic	5'-UUCUCCGAACGUGUCACGUTT-3'
miR-138 mimic	5'-AGCUGGUGUUGUGAAUCAGGCCG-3'
Control LNA	5'-CAGUACUUUUGUGUAGUACAA-3'
miR-138 LNA	5'-CGGCCUGAUUCACAACACCAGCU-3'

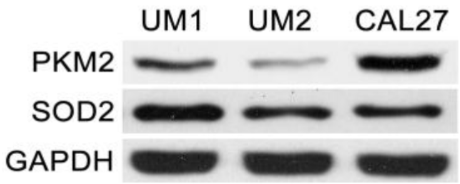

Supplementary Figure 1: Expression levels of PKM2 and SOD2 in TSCC cells with different migratory/invasive potentials as determined by Western blot analysis.

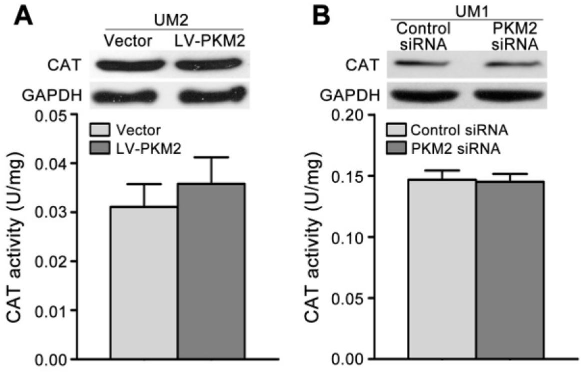

Supplementary Figure 2: Catalase (CAT) expression levels and activity in TSCC cell lines after PKM2 overexpression or knockdown. **(A)** CAT protein levels and activity did not change after PKM2 overexpression in UM2 cells. **(B)** CAT protein levels and activity did not change after PKM2 knockdown in UM1 cells. Vector: control lentiviral construct; LV-PKM2: lentiviral construct containing PKM2 cDNA.

Supplementary File 1.1: Microarray data of UM1 and UM2 cells.

See Supplementary File 1

Supplementary File 1.2: Microarray data of miR-138 transfected TSCC cells.

See Supplementary File 2
